# On the temporal interpretation of certain surprise questions

**DOI:** 10.1186/s40064-016-2951-5

**Published:** 2016-08-22

**Authors:** Alessandra Giorgi

**Affiliations:** Department of Linguistics and Comparative Cultural Studies, Ca’ Bembo, Dorsoduro 1075, 30123 Venice, Italy

**Keywords:** Surprise questions, Exclamations, Imperfect, Discourse grammar

## Abstract

This article considers a special kind of surprise questions, i.e. those introduced by the adversative particle *ma* (but), and compares it with surprise exclamations. The main issue addressed here concerns the obligatory presence in the questions of the imperfect verbal form, versus the obligatory presence in exclamations of a non-imperfect indicative. It will be shown that the special semantics associated with these structures determines the presence of a certain verbal form. Some syntactic issues will be addressed in the final section, having to do with the representation in the syntax of properties connected to the context.

## Background

In this paper I consider some counter-expectational surprise questions and exclamations in Italian, focusing in particular on the nature of tenses and temporal relations appearing in these structures. I will show that there is a systematic contrast between the cases with the imperfect and the sentences featuring other forms of the indicative. Such a contrast can be easily accounted for in a framework assuming Giorgi’s (2010) hypotheses concerning the representation of the speaker’s temporal coordinates in the sentence.^1^ I’ll also briefly discuss a syntactic representation for these sentences able to account for the position of the adversative particle *ma* (but). The goal of this work is twofold: on one hand it aims at clarifying the properties of these structures and their relation to the context, on the other it contributes to a deeper understanding of the properties of verbal forms, and in particular of the Italian, and more generally Romance, imperfect.

This article is organized as follows: in “The problem” section I illustrate the data and their main properties. In “The imperfect” section I sketch a brief account of the distribution of the imperfect versus the other forms of the indicative in Italian. In “On the temporal anchoring of the imperfect” section, I discuss the properties of temporal anchoring, with special reference to the imperfect, and then go back to the discussion of the counter-expectational constructions. In “Further issues” section I explore some further issues related to their syntactic structure.

## The problem

### The data

Here I analyze some examples, beginning with surprise questions. Consider the following scenario: Mary calls me on the phone and tells me that she has a fine new red dress to wear at tonight’s party. When I meet her at the party, I see that she has a blue gown. I might then ask:

(1): (Ma) non era rosso?
  (But) not was-IMPF red
  ‘(But) wasn’t it red?’

Where the adversative particle *ma* (but) is optional.^2^

Let’s compare these examples with exclamations.^3^ Imagine the following scenario: Mary informs me that she is going to buy her wedding dress. Later she shows me her purchase and I see that it is a red gown, an unusual color for this kind of dress. I may react by saying:^4^

(2): (Ma) è rosso!
  (But) it’s red!

These sentences exhibit several interesting properties. They are in both cases associated with a characteristic intonation and are introduced—or can be introduced—by the particle *ma*, which in normal cases cannot introduce main clauses or interrogatives. Moreover, these sentences cannot be embedded, with or without the complementizer *che* (that):^5^

(3): *Gianni ha detto che ma è rosso
  Gianni said that but it is red
(4): *Gianni ha detto ma è rosso
  Gianni said but it is red
(5): *Gianni ha detto che ma non era rosso
  Gianni said that but it wasn’t-IMPF red
(6): *Gianni ha detto ma non era rosso
  Gianni said but it wasn’t-IMPF red

The particle *ma* (but), which I briefly analyze in “Further issues” section, is an adversative coordinating particle. Without entering in much detail, let me just point out that all these sentences are counter-expectational, in that the speaker expresses her surprise at a state of things different from what she expected. The contribution of *ma* to yield this effect is crucial.

### The verbal form

Let’s consider example (1). As proposed above, the particle *ma* signals the counter-expectational character of the utterance, which is associated to the interrogative intonation. Analogously, in example (2), *ma* signals counter-expectation in association with an exclamative intonation. Interestingly, in these pairs a surprise negation appears—cf. Delfitto and Fiorin (2014a, b).^6^

Here I focus on the analysis of the verbal forms: the present indicative in (1) and the imperfect in (2). Note that the usage of the present tense would be inappropriate in the situation described above for (1):

(7): #Ma non è rosso?
  But isn’t it red?

Sentence (7) would not be appropriate if the speaker has the expectation of a red dress and sees a dress of another color. Sentence (7) is not ungrammatical, in the sense that it can still be a rhetorical/surprise question to be uttered for instance in the following situation: Mary, pointing to a dress exposed in a window, tells Paul: “How beautiful that blue dress!” and Paul might answer: “But isn’t it red?”, because he is seeing it as red and not as blue. Hence, for the speaker in this case the redness of the dress is a fact, whereas in the case of example (1) it is an expectation.

The conditions for the appropriateness of the sentence with the present tense, therefore, are quite different with respect to those holding for the sentence with the imperfect. By means of (1), the utterer wants to convey the meaning that the color he is seeing—blue in this case—is not what he was expecting, namely, red. On the other hand, in the situation described for (7), Mary’s remark leads to a comparison between the state of things implied by her words—the dress is blue—and Paul’s perception that the dress is red.

Conversely, the usage of the imperfect in a situation where sentence (3) is appropriate yields unfelicitous results:

(8): #Ma era rosso!
  But it was-IMPF red!

By uttering (8) the speaker wants to convey the idea that the dress she is looking at is not red at that time, but it was red at some previous time, implying that in some way it changed color, for instance by dying.

Concluding this section, note that it is not the case that the non-imperfect indicative cannot be used in surprise questions or exclamations, is just that it is inappropriate in the scenario described for (1). The opposite holds of example (2). This shows that the presence of the imperfect versus the non-imperfect indicative is crucial in conveying the special meaning the speaker wants to express by means of these sentences. In what follows I propose an explanation for this fact.^7^

## The imperfect

In this section I’ll briefly illustrate the properties of the imperfect that are relevant for the issue addressed in this work.

### The imperfect as an anaphoric verbal form

The imperfect can have both temporal readings and non-temporal ones. Consider the following examples:^8^

(9): #Mario mangiava una mela.
  Mario ate(IMPF) an apple.
(10): Alle tre Mario mangiava una mela
  At three Mario ate(IMPF) an apple

As signaled by the diacritic, example (9) is odd if uttered out-of-the-blue, i.e. in absence of a previous context, either provided in the sentence or in the discourse. The contrast with example (10) shows that the imperfect requires a temporal reference, in this case provided by the temporal locution *alle tre* (at three). The temporal reference can also be provided in the previous discourse, as in the following case:

(11): Cosa faceva Mario alle tre?
  What was Mario doing at three?
(12): Alle tre Mario mangiava una mela
  At three Mario ate(IMPF) an apple

Furthermore, the ungrammaticality of the example in (14) shows that in these cases the imperfect is interpreted as a past tense:

(13): Quando Gianni è uscito, Maria guardava la TV
  When Gianni left, M. was watching(IMPF) TV
(14): *Quando Gianni uscirà, Maria guardava la TV
  When Gianni will leave, Maria was watching(IMPF) TV

Examples (13) and (14) show that a *when* clause can provide the required temporal reference to the imperfect—cf. (13)—but it cannot do so if the intended time of the event is in the future, as in (14). Hence, in these cases the imperfect contributes a past temporal value, in this work, however, I will not further address the issues related to the past temporal value of the imperfect and refer the reader to the references given above.

### The imperfect as a modal verbal form

There are cases in which the imperfect is not interpreted as a past. This verbal form in Italian can also co-occur with future-oriented temporal phrases:^9^

(15): Mario partiva domani.
  Mario left(IMPF) tomorrow.
(16): *Mario è partito/partì domani.
  Mario has left/left tomorrow.

Example (15) is not a simple assertion concerning an event occurring in the future. It has a special modal meaning, a possible paraphrase is the following:

(17): Mario had the intention/was committed to leave tomorrow

The contrast between examples (15) and (16) shows that this property is a peculiarity of the imperfect and not a general feature of past temporal forms in Italian. Both the present perfect and the simple past in example (16) give rise to ungrammaticality with a future temporal reference such as *domani* (tomorrow). In what follows I’ll provide examples only with the present perfect, which is my native variant.

### The imperfect in fictional contexts

The imperfect obligatorily appears in *fictional* and *dream* contexts. From the point of view of temporal relations, the notion of *pastness* in these contexts applies in a different way with respect to normal ones.

Consider in the first place the so-called *imperfâit preludique*, typically used by children while planning a new game (cf. Vet 1983):

(18): Facciamo che io ero il re e tu la regina
  Let’s pretend that I am(IMPF) the king and you the queen

In these contexts the embedded verbal form is used to express a plan and does not refer to a past event. The same happens in the case of stage instructions. Imagine a scenario where a director explains a scene to an actor:

(19): A questo punto Mario usciva e tu lo seguivi.
  At this point Mario left(IMPF) and you followed(IMPF) him.

In this case as well the embedded imperfect is not referring to a past event. Note also that the presence of the imperfect is obligatory, as shown by the following examples:

(20): #Facciamo che io sono stato/fui il re e tu la regina.
  Let’s pretend that I was(PRES PERF) the king and you the queen.
(21): #A questo punto Mario uscì e tu lo seguisti.
  At this point Mario left(PAST) and you followed(PAST) him.

The usage of the present perfect or of the simple past in these contexts yields infelicitous sentences.

Furthermore, the imperfect is the most common form in narrative contexts, such as e.g. story-telling, fictions, etc., as in the cases like the following one:

(22): Il ladro passeggiava nervosamente. Qualcosa era andato storto… [from Giorgi and Pianesi (2001, ex. 53)]
  The thief walked(IMPF) nervously. Something had(IMPF) gone wrong…

Example (22) could be the beginning of a novel. Note that there is no given temporal reference in this case, contrasting with examples (10) above.

Finally, the imperfect is the verbal form appearing in Italian in the contexts embedded under the verb *sognare* (dream):

(23): Mario ha sognato che Carlo vinceva alla lotteria.
  Mario dreamed that Carlo won(IMPF) the lottery.

In these cases, the imperfect tense does not have any temporal value, in that it does not contribute to locate the eventuality with respect to the utterance time—where the utterance time ultimately coincides with the speaker’s temporal location—or any other temporal anchor, as I’ll discuss with more details in the next section. The winning of the lottery in (23) is the content of the dream and as such, it is not located in the past present or future with respect to the dream itself. I.e. it is not the case that Mario dreamed of an event located in his past. In this sense, example (23) contrasts with the following example:

(24): Mario ha detto che Carlo ha vinto alla lotteria.
  Mario said that Carlo won(PRES PERF) the lottery

In (24) Mario reported an event—the winning of the lottery by Carlo—located in his own past. As we will see below, in this case the embedded event is anchored, whereas in the preceding one it is not. Moreover, notice that whereas in (23) the imperfect is the form which is normally used, the same form in (24) would be odd, on a par with example (9) above:

(25): #Mario ha detto che Carlo vinceva alla lotteria.
  Mario said that Carlo won(IMPF) the lottery

The only natural interpretation for (25) is a habitual one, as illustrated in the following example:

(26): Mario ha detto che Carlo vinceva alla lotteria ogni volta che giocava
  Mario said that Carlo won(IMPF) the lottery every time he played

In (26), where the locution *every time he played* selects the habitual reading, the usage of the imperfect is appropriate. These considerations do not apply to the fictional contexts presented above.

## On the temporal anchoring of the imperfect

### A brief discussion of temporal anchoring

In this section I briefly sketch the basic notions concerning temporal anchoring in complement clauses that will be needed in this work, capitalizing on the hypotheses discussed in Giorgi and Pianesi (1997) and Giorgi (2010) and then I’ll go back to the distribution and properties of imperfect verbal forms.^10^

Let’s consider a simple structure with a subordinate clause including an eventive predicate:

(27): Gianni ha detto che Maria ha mangiato un panino
  Gianni said that Maria ate (PRES PERF) a sandwich
(28): Gianni ha detto che Maria mangerà un panino
  Gianni said that Maria will eat(FUT) a sandwich

Enç (1987) argued that temporal anchoring is obligatorily required. Hence, the embedded event must be temporally located with respect to the main one. In example (27) the embedded clause features a past verbal form and example (28) a future one. The embedded past predicate is interpreted as preceding the main *saying* predicate and analogously the embedded future predicate is interpreted as future with respect to the main predicate of *saying*.

However, note that in (28) the embedded future event must be future also with respect to the utterance time. I.e. it cannot be the case that the *eating* event is future with respect to the *saying* and past with respect to the speaker’s utterance. Hence, in this case the embedded event must be located in the future both with respect to the main event and to the utterance one. In Italian, and in many other languages as well, in order to express this particular temporal relation, the so-called future-in-the-past must be used, as in the following case:

(29): Gianni ha detto che Maria avrebbe mangiato un panino
  Gianni said that Maria would eat a sandwich

In Italian the *would*-future is expressed by means of a perfect conditional.^11^ By means of this form, the speaker does not commit herself to an interpretation of the embedded event as future with respect to the utterance time, but only to one where the eating is future with respect to the main event.

Consider also that the *double* interpretation of the embedded event in (28) is *a fortiori* assigned to the embedded past event in example (27). Here the eating is past with respect to the saying, which is itself located in the past with respect to the utterance time, hence the eating is necessarily in the past with respect to the utterance time as well.

How can the double interpretation of the embedded future be accounted for? Giorgi and Pianesi (1997) argue in favor of a *generalized Double Access Reading theory*.^12^ The basic example for the DAR includes a present tense embedded under a past verbal form:

(30): Gianni ha detto che Maria è incinta
  Gianni said that Maria is pregnant

For this sentence to be felicitous, the state of pregnancy must hold both at the time of the saying and at utterance time. Consider in fact that if that is not possible, due to the intrinsic properties of the predicates appearing in the sentence, the structure is infelicitous:

(31): #Due anni fa, Gianni ha detto che Maria è incinta
  Two years ago, Gianni said that Maria is pregnant

Given what we about the state of pregnancy, in this case Maria cannot be pregnant both at the time of saying and at the time of the utterance. Hence, the sentence is ruled out.

Giorgi and Pianesi (1997) argued that the DAR is not just a property of an embedded present tense, but a general property of embedded contexts, in particular when they feature a present, past (realized as a present perfect in Central/Northern Italy) or future indicative. In other words, in examples (27), (28) and (30), the embedded eventuality must be interpreted twice: once with respect to the superordinate predicate and once with respect to the utterance event, i.e. with respect to the speaker’s temporal location. Therefore according to Giorgi and Pianesi, the DAR is a general property of the anchoring process, when applied to present, past and future indicative forms.

Giorgi (2010) argues that the anchoring of the indicative form to the utterance time is a combined effect of the morpho-syntax of the indicative present, past and future, on the one hand, and, on the other, of the syntactic structure of the clause embedded under a verb of saying—or more generally, under a verb requiring an indicative verbal form, as opposed for instance to a subjunctive one. Giorgi (2010) proposes that these verbal forms are indexical, in the sense that they require feature checking with the speaker’s temporal location. The speaker’s temporal location is syntactically realized in the specifier position of the highest projection in the C-layer of the verbs selecting indicative clauses.^13^ The checking of the verbal features is obligatory, hence it is the presence of those particular temporal forms which gives rise to the Double Access Reading.

### Temporal anchoring and the imperfect

Note that no (generalized) double access reading obtains with an embedded imperfect:

(32): Gianni ha detto che Maria era incinta
  Gianni said that Maria was(IMPF) pregnant

In this case the state of pregnancy is simultaneous with the saying and does not have to hold at the time of the utterance, to the extent that the following sentence contrasts with sentence (31) above:

(33): Due anni fa, Gianni ha detto che Maria era incinta
  Two years ago, Gianni said that Maria was(IMPF) pregnant

In example (33) the state of pregnancy cannot extend up to the utterance time, still the sentence is fine. This means that the imperfect does not share the indexicality requirement of the other indicative forms, i.e. its morpho-syntax does not include the features that must be checked by those associated to the speaker’s projection in the highest spec of the C-layer.^14^

This fact is not surprising, given the consideration above concerning its anaphoricity. The examples (9) and (10) above, reproduced here for clarity, showed that in fact the imperfect *cannot* be anchored to the utterance time:

(34): #Mario mangiava una mela.
  Mario ate(IMPF) an apple.
(35): Alle tre Mario mangiava una mela
  At three Mario ate(IMPF) an apple

The imperfect always requires a temporal reference provided by the context and cannot be directly anchored to the speaker’s temporal location. Hence, technically, in this case the imperfect is anchored by the temporal locution, which on its turn is anchored to the indexical context.

Let’s go back to the cases discussed above. The imperfect can be compatible with a future temporal reference such as *domani* (tomorrow), as illustrated in example (15) above, precisely because it is not an indexical verbal form and it is not a priori specified as present, past or future with respect to the speaker’s temporal location. Coherently with these considerations, the imperfect is the verbal form used in fictional contexts of various sorts, as showed by the examples in (18), i.e. the *imperfait preludique*; (19), i.e. stage instructions; (22), i.e. narrative contexts and finally (23), i.e. *dream* contexts. In all these cases, the imperfect event is not anchored, in that it is not interpreted as past with respect to the utterance time. In other words, the very notion of past-ness in these cases does not apply, at least not as in normal cases.

### Back to counter-expectational surprise questions and exclamations

Let’s go back to examples (1) and (2), reproduced here for clarity:

(36): (Ma) non era rosso?
  (But) not was-IMPF red
  ‘(But) wasn’t it red?’
(37): (Ma) è rosso!
  (But) it’s red!

I have shown in examples (7) and (8), also reproduced here, that the usage of the present in (36) and the imperfect in (37) would yield infelicitous results in those contexts felicitously admitting (36) and (37):

(38): #Ma non è rosso?
  But isn’t it red?
(39): #Ma era rosso!
  But it was-IMPF red!

On the basis of the arguments discussed above it is possible to provide an explanation for this contrast. In the contexts where (36) is appropriate, the embedded verbal form cannot be anchored to the indexical context, in that the redness of the dress is not a fact, but belongs to the realm of the speaker’s expectations. The sentence is counter-expectational precisely because the dress is indeed *not* red. In other words, *to be red* in this case cannot be anchored to the utterance event, because the predicate does not refer to the context where the speaker is temporally located. Hence, only a verbal form admitting a non-indexical anchoring, such as the imperfect, can appear in these contexts. The indicative in fact forces anchoring to the speaker’s temporal location, as emerges from the discussion of example (38) provided in “The problem” section. In that case, for the sentence to be appropriate, the dress must indeed be red and the surprise, when the sentence entails it, stems from other considerations. The opposite is true in the case of the exclamative sentence in (37). The surprise feeling is due to the very fact that the dress is red, hence an indexical verbal form is required and, conversely, an imperfect would be infelicitous.

## Further issues

In this section I briefly address some additional issues related to this sentence, in particular the syntax of the particle *ma.* I do not intend to provide a full discussion, which would necessitate further work, but only to sketch some possible line of future research.

I already pointed out in “The problem” section that these clauses cannot be embedded; here I consider further syntactic properties. The interrogative constructions are compatible with a clitic left dislocated (CLLD) phrase, only when it appear on the right of *ma*:^15^

(40): Ma a Gianni, non gli avevi comprato il gelato?
  But to Gianni, not to him(CL) (you) had(IMPF) bought the ice-cream?
  ‘didn’t you bought ice-cream to the kids?’
(41): ??A Gianni, ma non gli avevi comprato il gelato?
  To Gianni, but not (you) to him(CL) had(IMPF) bought the ice-cream?
  ‘To Gianni, didn’t you bought ice-cream?’

A CLLD can follow *ma*, as shown by example (40), but is much less acceptable when it appears on its left, as shown by example (41).

In the exclamative clauses, the situation is almost the same, with the only difference that the CLLD on the left of *ma* is even worse:

(42): Ma a Gianni, gli hai comprato il gelato!
  But to Gianni (you) to him bought ice-cream!
  ‘But you bought ice-cream to Gianni!’
(43): *A Gianni ma gli hai comprato il gelato!
  To Gianni but (you) to him bought ice-cream!
  ‘But you bought ice-cream to Gianni!’

Consider now the distribution of contrastive focus (in capital letters):

(44): *Ma IL GELATO (non la torta) non avevi comprato a Gianni?
  But THE ICE-CREAM(not the cake) (you) not had bought to Gianni?
  ‘but hadn’t you bought the ice-cream to Gianni (not the cake)?’
(45): *IL GELATO ma non avevi comprato a Gianni?
  THE ICE-CREAM but (you) not had bought to Gianni
  ‘but hadn’t you bought the ice-cream to Gianni?’

In this case a contrastive focus is ungrammatical. This is however expected, given that in general, as is well-known, in Italian this kind of focus is incompatible with questions.

Exclamatives however do not partake of this restriction, so that a contrastive focus following *ma* is grammatical:

(46): Ma IL GELATO hai comprato! (non la torta)
  But the ICE-CREAM you bought! (not the cake)
  ‘But you bought ice-cream! (not the cake)’

Still, the focused phrase cannot precede the particle:

(47): *IL GELATO ma hai comprato! (non la torta)
  the ICE-CREAM but you bought! (not the cake)
  ‘But you bought ice-cream! (not the cake)’

Giorgi (2015) argues that a focused phrase is internal to the sentence and represented syntactically presumably in the left periphery, as proposed by Rizzi (1997). A CLLD on the contrary, is totally external, and is much more similar to a parenthetical construction. An important argument in favor of this view is that contrastive focus phrases are moved, whereas CLLD ones seem to be base generated.^16^

The incompatibility of contrastive focus with these constructions introduced by *ma,* therefore, points to the conclusion that there is no suitable landing site on its left. Moreover, even if CCLD is base generated, it is still not acceptable—or at most very marginal—with these sentences. Note that this effect cannot be due to semantic incompatibility, because both contrastive focus and CLLD are acceptable when they follow *ma.*

Furthermore, consider also that a hanging topic on the left of *ma* is fully acceptable in both constructions:

(48): Gianni, ma non gli avevi comprato un gelato?
  Gianni, but (you) non to him(cl) had(IMPF) bought an ice-cream?
  ‘Gianni, but didn’t you bought him an ice-cream?’
(49): Gianni, ma gli hai comprato un gelato!
  Gianni, but (you) to him(cl) bought an ice-cream!
  ‘Gianni, but you bought him an ice-cream!’

Example (48) contrasts with the CLLD one given in (41).

Giorgi (2015) proposes that the hanging topic is still more external to the sentence it refers to, than a CLLD phrase. Capitalizing on an observation by Cinque (2008), Giorgi (2015) proposes that the hanging topic and the following sentence, though apparently constituting a single sentence, are actually a discourse, formed by two separated components: the sentence and the hanging phrase. The two components are connected by a silent head; the Hanging phrase occupies its specifier position, and the sentence its complement one. Consider for instance the following example:^17^

(50): Gianni, Maria gli ha fatto un bellissimo regalo
  Gianni, Maria to him-cl has given a wonderful present
  ‘Gianni, Maria gave him a wonderful present’

According to hypotheses argued for in Giorgi (2015), the structure assigned to it is the following:

(51): _DISCOURSE_ Gianni dis Maria gli ha fatto un bellissimo regalo
  Gianni, Maria to him-cl has given a wonderful present

Where the head dis connects the two parts of the discourse.

Going back to the sentences discussed in this work, I would like to propose here that in these cases, the head DIS is realized by the particle *ma*, giving rise to the following structures:

Where *ma* is the head of a discourse containing the interrogative clause as its complement. Analogously, the exclamative sentence can be represented as follows:

If the interrogative or exclamative sentence is the complement of the adversative particle, what constitutes the rest of discourse? My suggestion, to be further investigated, is that the dots under the specifier actually represent the expected, but silent, portion of the construction, namely in this case, the expectation for the dress to be, or to be not, red.^18^

Going back to example (40)–(49), the incompatibility with contrastive focus on the left of *ma*, is explained by the simple consideration that syntactic movement does not move *beyond* the single sentence. Since contrastive focus is the result of a movement derivation, it is incompatible with this kind of constructions.

Hanging topic, on the contrary, as illustrated above, gives rise to a discourse as well. Hence, for a sentence such as (48), the structure would be the following:

In (54) only the lower discourse head is realized, by means of *ma*, the higher one, heading the projection hosting the hanging topic, is silent. The marginality of CLLD, according to this hypothesis, is due to the fact that when on the left of *ma*, it is supposed to appear in the same position as the hanging topic, which might create a problem, since the two constructions are very similar, but not identical. On this issue, I refer the reader to the discussion in Giorgi (2015).

Let me add two more brief considerations. As noted in “The problem” section, the particle *ma* can be omitted. Under the hypothesis sketched here, this is not surprising, since discourse heads are usually silent. When silent, the adversative meaning must be uniquely supplied by the context.

The second consideration concerns the presence of negation in the interrogative sentences. Delfitto and Fiorin (2014b), point out that negation in surprise exclamatives is expletive, i.e. it does not contribute its canonical meaning as a propositional operator. The same can certainly be argued with respect to the surprise interrogatives discussed here. However, note that the presence of the negation is these cases goes together with the falseness of the predicate in the actual world, e.g. in the case presented in (1), the dress is in fact *not red.* Hence, there is a sense under which the negation indeed negates the predicate. The significance of this observation must be ascertained by means of further research.

## Conclusions

In this article I investigated some properties of counter-expectational surprise questions and exclamatives in Italian. I argued that the presence of the imperfect in the former constructions is due to the peculiar morpho-syntactic properties of the imperfect. In fact, only the imperfect in the Italian temporal system can be compatible with the interpretation that is assigned to these constructions. I also briefly discussed some syntactic properties, showing that a multi-sentential—in terms of *discourse*—analysis can account for a broader range of facts than a simple one-sentence structure. Further research is indeed necessary especially with respect to other important issues, such as for instance the presence of negation in sentences like (1).

## Footnotes

1 The term counter-expectational is used by Vicente (2010), who addresses a similar issue in Spanish.

2 According to my native judgment, the presence of the particle *ma* is *almost* obligatory, meaning that its absence does not automatically makes the sentence ungrammatical, but it does make it odd. I’ll be back on this issue in “Further issues” section below.

3 Exclamatives have been recently studied by several scholars, among the others, see Portner and Zanuttini (2003), and Delfitto and Fiorin (2014a, b); for a very peculiar construction, very likely related to the one discussed here, see Poletto and Zanuttini (2013) on *sì che*/*no che* (yes that/no that) constructions. In this paper I do not have anything to add to Poletto and Zanuttini’s discussion, given that I’m addressing a very particular issue, i.e. the one concerning the presence of the imperfect versus the indicative.

4 The same holds with respect to non-copular structures as well. Consider for instance the following scenario. In the morning, John runs the marathon and his wife calls me to tell me that he won. Later that night, I meet John and he tells me that he arrived second. I may ask:

i.: (Ma) non avevi vinto? (But) not (you) had-IMPF won? ‘(But) hadn’t you won?’

With respect to the exclamative case, consider the following scenario: I know that John is not a strong runner and that he is going to participate to the marathon, even if it is very unlikely he will ever win. When the next day I read on the newspaper that he won, I may say:

ii.: (Ma) ha vinto! (But) he has won! ‘(But) he won!’

5 The particle *ma* is not a complementizer, hence it cannot appear in subordinate clauses with that role. Irrelevantly, examples (4) and (6) would be acceptable if interpreted as quotations:

i.: Gianni ha detto: “Ma è rosso!” Gianni said: “But it is red!”
ii.: Gianni ha detto: “ma non era rosso?” Gianni said: “But wasn’t(IMPF) it red?”

6 See also a very brief discussion in “Further issues” section.

7 Note also that negation is present in (1), but not in (2). I will briefly consider this issue below in “Further issues” section.

8 For extensive discussion of the Italian imperfect, see Giorgi and Pianesi (1997 and 2001). See also, for different perspectives, Delfitto and Bertinetto (1995), Delfitto (2004), Ippolito (2000).

9 In Italian the distribution of the present and the simple past is geographically determined: in Central and Northern Italy speakers normally use the present perfect, in Southern Italy the simple past.

10 There is an extensive and important literature on temporal anchoring which will be impossible to review here. Let me just mention, among the many others working in the generative framework, Zagona (1992, 2003, 2007), Stowell (1996), Demirdache and Uribe-Etxebarria (2000), Guéron (2007). All these approaches present advantages and disadvantages with respect to the one argued for here, which I’m not able to address in this work. However, for the particular point I want to make here, a general discussion of the framework involved does not seem to be crucial.

11 On this point, see the discussion in Giorgi and Pianesi (1997).

12 On the Double Access Reading see, among the many others, Ogihara (1995), Abusch (1997), Sharvit (2003), Schlenker (2003).

13 In Italian verbs of saying and of communication select an embedded indicative; verbs of thinking, believing, wishing, hoping etc. select a subjunctive. No double access reading obtains with a subjunctive embedded form. See Giorgi and Pianesi (1997) and Giorgi (2010) for a more detailed analysis.

14 It is possible to show that the imperfect is indeed an indicative form, as argued by Giorgi and Pianesi (1997). As a test, we can consider the distribution of complementizer deletion—i.e. the omission of the complementizer *che* (that). In Italian it is possible to delete the complementizer *che* when introducing subjunctive clauses, whereas this is not possible with the indicative ones. The imperfect verbal forms pattern with the indicative.

15 In Italian a clitic left dislocated phrase as the one appearing in example (40) is informationally given.

16 There is an extensive discussion on this point. See among the others Cinque (1999) and Frascarelli (2000). The authors discuss hanging topic as well, showing that even this kind of phrases are base generated and not moved in the position they occupy.

17 See also Giorgi (2016) for a discussion of the same framework, with respect to the parentheticals introducing free indirect discourse and quotations.

18 Consider that the complement of the discourse head is certainly a CP, i.e. a maximal sentence. In fact it is possible to have a sentence introduced by *che* (that) following *ma*:

i.: Ma che hai comprato il gelato? But that you bought the ice-cream? ‘but did you bought ice-cream?’
